# Usefulness of High Suction Pressure for Sufficient Tissue Collection During Endobronchial Ultrasound Guided Transbronchial Needle Aspiration

**DOI:** 10.1371/journal.pone.0082787

**Published:** 2013-12-05

**Authors:** Takayuki Shiroyama, Norio Okamoto, Hidekazu Suzuki, Motohiro Tamiya, Tadahiro Yamadori, Naoko Morishita, Tomoyuki Otsuka, Satomu Morita, Kanako Kurata, Akira Okimura, Kunimitsu Kawahara, Shinji Sasada, Tomonori Hirashima, Ichiro Kawase

**Affiliations:** 1 Department of Thoracic Malignancy, Osaka Prefectural Medical Center for Respiratory and Allergic Diseases, Osaka, Japan; 2 Department of Pathology, Osaka Prefectural Medical Center for Respiratory and Allergic Diseases, Osaka, Japan; 3 Department of Pathology, Steel Memorial Hirohata Hospital, Hyogo, Japan; 4 Department of Endoscopy, Respiratory Endoscopy Division, National Cancer Center Hospital, Tokyo, Japan; University of British Columbia, Canada

## Abstract

**Introduction:**

The optimal suction pressure during endobronchial ultrasound guided transbronchial needle aspiration (EBUS-TBNA) remains to be determined. The aim of this study was to compare suction pressures for performance in collecting sufficient tissue specimens from mediastinal and hilar lymph nodes during EBUS-TBNA.

**Methods:**

Retrospective analysis of consecutive patients with mediastinal and hilar lymphadenopathy who underwent EBUS-TBNA over a 3-year period. Results from patients who underwent EBUS-TBNA using a dedicated 20-mL VacLoc (Merit Medical Systems, Inc, South Jordan, UT) syringe (conventional method, group C) were compared with results from patients in whom a disposable 30-mL syringe (high pressure group, group H) was used. The yield for sufficient histologic specimen retrieval and amount of tissue obtained were compared between the 2 groups.

**Results:**

Of 178 patients who underwent EBUS-TBNA, 131 had lung cancer confirmed by EBUS-TBNA: 35 in group C and 96 in group H. There were 7 patients in group C and 6 in group H who received final diagnoses by cytology alone. There were 28 in group C and 90 in group H who were diagnosed by both cytology and histology. There was a statistically significant difference between the groups in terms of the rate of sufficient sampling for histological specimens (*p* = 0.04). The H group revealed a tissue area approximately twice that of the C group (*p* = 0.003). There were no major procedure-related complications in either group.

**Conclusion:**

Higher suction pressures with larger syringe volumes during EBUS-TBNA may be useful for safely collecting sufficient tissue specimens.

## Introduction

Endobronchial ultrasound-guided transbronchial needle aspiration (EBUS-TBNA) is a safe, minimally invasive diagnostic modality with a high yield for the diagnosis of mediastinal and hilar lymphadenopathy. Cytological and histological samples can be obtained by EBUS-TBNA, therefore facilitating comprehensive evaluations such as immunohistochemistry and mutation analysis. EBUS-TBNA can provide the quality and quantity of aspirates important for genetic analysis such as epidermal growth factor receptor (EGFR) mutation or anaplastic lymphoma kinase (ALK) translocation in lung cancer patients [1–3]. The aim of this study was to assess the usefulness of high suction pressure for tissue aspiration during EBUS-TBNA. At present, the optimal aspiration pressure for specimen collection by EBUS-TBNA remains unclear.

## Patients and Methods

### Patients

We retrospectively examined 178 consecutive patients who underwent EBUS-TBNA for diagnosis of mediastinal and hilar lymphadenopathy at our institute from April 2009 to March 2012. The study was approved by the institutional review board at the Osaka Prefectural Medical Center for Respiratory and Allergic Diseases. Requirement for informed consent was waived by the committee for this retrospective analysis of clinical data and the data were analyzed anonymously. Data were obtained from paper and electronic medical records, and patients were divided into 2 groups according to the volume of suction pressure used for specimen collection during EBUS-TBNA. Patients who underwent EBUS-TBNA using the dedicated 20-mL VacLok syringe were in group C (conventional method), and those in whom a disposable 30-mL syringe was used were in group H (high pressure). The rates of sufficient histologic specimen retrieval and diagnostic yields were compared between the 2 groups.

### EBUS-TBNA technique

EBUS-TBNA was performed with a convex probe EBUS (CP-EBUS; BF-UC260FW, Olympus, Tokyo, Japan) using dedicated 22-gauge needles (NA-201SX-4022, Olympus, Tokyo, Japan) in all cases; a bronchoscopist who had been previously trained in EBUS-TBNA techniques on training mannequins and animal models performed the procedure under the supervision of experts. All patients were under moderate sedation with intravenous midazolam during the procedures.

**Figure 1:**
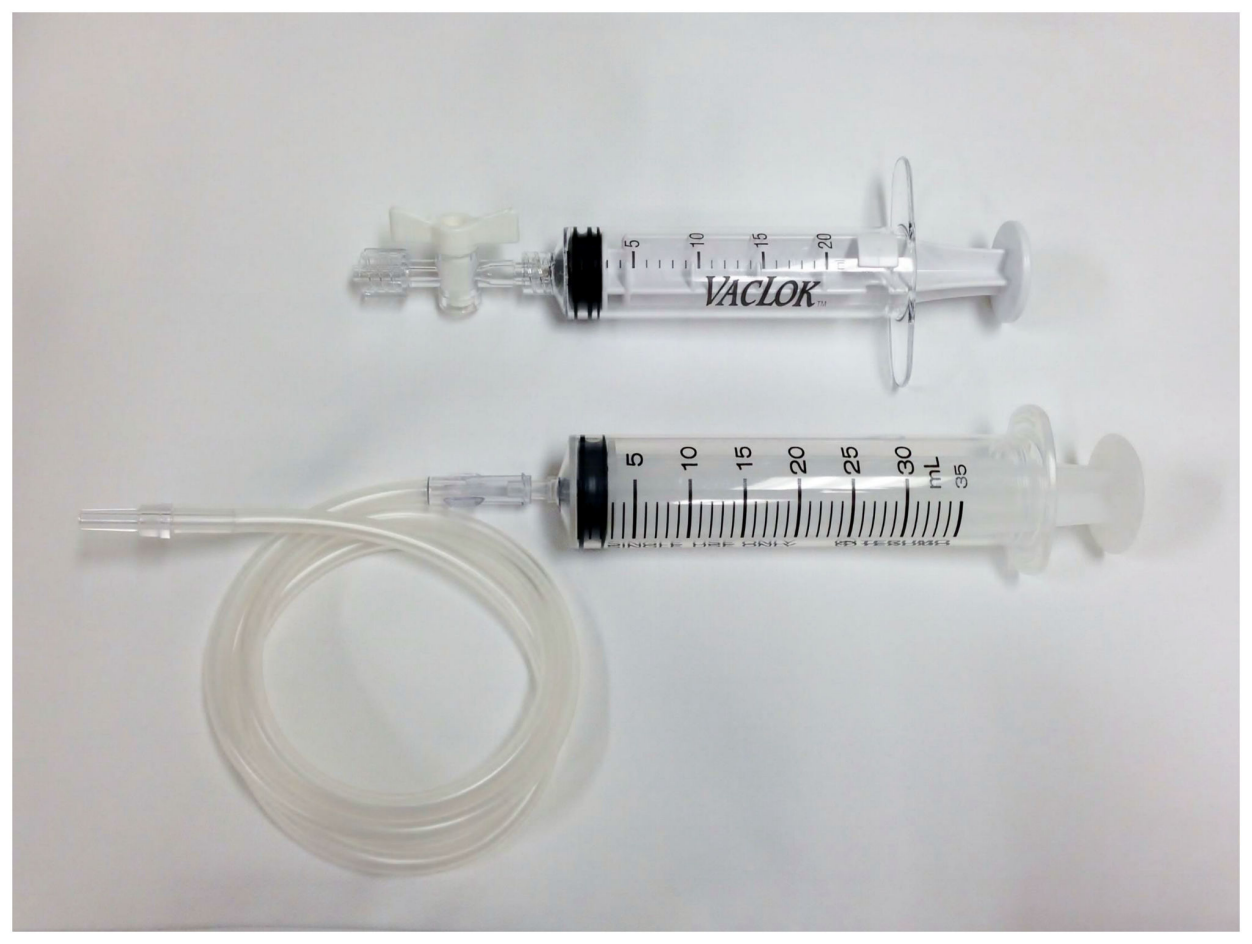
Suction pressure devices. The upper syringe is a dedicated VacLok 20 mL syringe, as used in group C, and the lower syringe is a disposable 30 mL syringe, as used in group H.

Two passes per lymph node were routinely performed in all cases. If these passes did not yield adequate material, only 1 more pass inside the same lymph node was additionally performed. If no histological core was macroscopically available, different lymph node aspiration was performed until up to 3 passes per lymph node. After lymph node puncture, the internal stylet was removed and suction pressure was applied with the syringe. Syringe pressure was applied during each aspiration to a volume of 20 mL in group C (conventional method, dedicated VacLok syringe) or to approximately 30 mL in group H (high pressure, disposable syringe) (Figure 1). In each puncture, the needle was to be moved back and forth inside the lesion of interest 10-15 times around 30 s during aspiration. In case blood entered the suction syringe during vascular lymph node aspiration, the pressure on the syringe was released immediately, and the needle was removed without any further passes at that point.

Finally, the needle was retrieved and the internal sheath replaced to push the histologic core specimen from the needle. After completing the puncture, the aspirated material could be smeared onto glass slides. Smears were air-dried and fixed in 95% alcohol for cytology. In cases where core tissues were obtained for histology, the specimens were pushed out of the needle onto a filter paper to absorb excess blood and eventually fixed in formalin for histological analysis. The aspirate remaining in the needle was rinsed into a tube containing normal saline and available for molecular testing if malignancies were confirmed. In our study, histologic specimen included only the tissue core and not the cell block. Rapid, on-site cytological examination (ROSE) was performed in all cases. Histologic cores were stained with hematoxylin and eosin. Immunohistochemistry was also performed if necessary.

### Data collection and evaluation

To assess the usefulness of high suction pressure aspiration during EBUS-TBNA, we distinguished between cases diagnosed only by cytological findings and those diagnosed by both cytological and histological findings with respect to lung cancer. The EBUS-TBNA was regarded as failed in collecting biopsy specimens if nothing was obtained by EBUS-TBNA or if pathologists found submitted specimens insufficient due to the lack of quality and quantity. To compare the amount of histologic tissue between the groups, we scanned the maximum surface area of the histologic samples using hematoxylin and eosin stained slides, counted the number of tissue fragments, and measured the total tissue area obtained with each technique. The measurements were performed using ImageJ software (version 1.47). If EBUS-TBNA did not provide a diagnosis, surgical biopsy or transbronchial biopsy followed. In this study, diagnoses of benign diseases were based on results of clinical follow up of at least 6 months demonstrating reduction in tumor size or a lack of apparent radiologic disease progression.

### Statistical analysis

The sensitivity, specificity, and rates of diagnostic accuracy were calculated by standard definitions. All statistical analyses were performed using software R (version 3.0.1). Continuous variables were analyzed using Mann–Whitney *U* test and categorical variables were analyzed using Fisher’s exact test. All *p* values were based on a two-sided hypothesis, and a *p* value of <0.05 was considered statistically significant.

## Results

**Figure 2:**
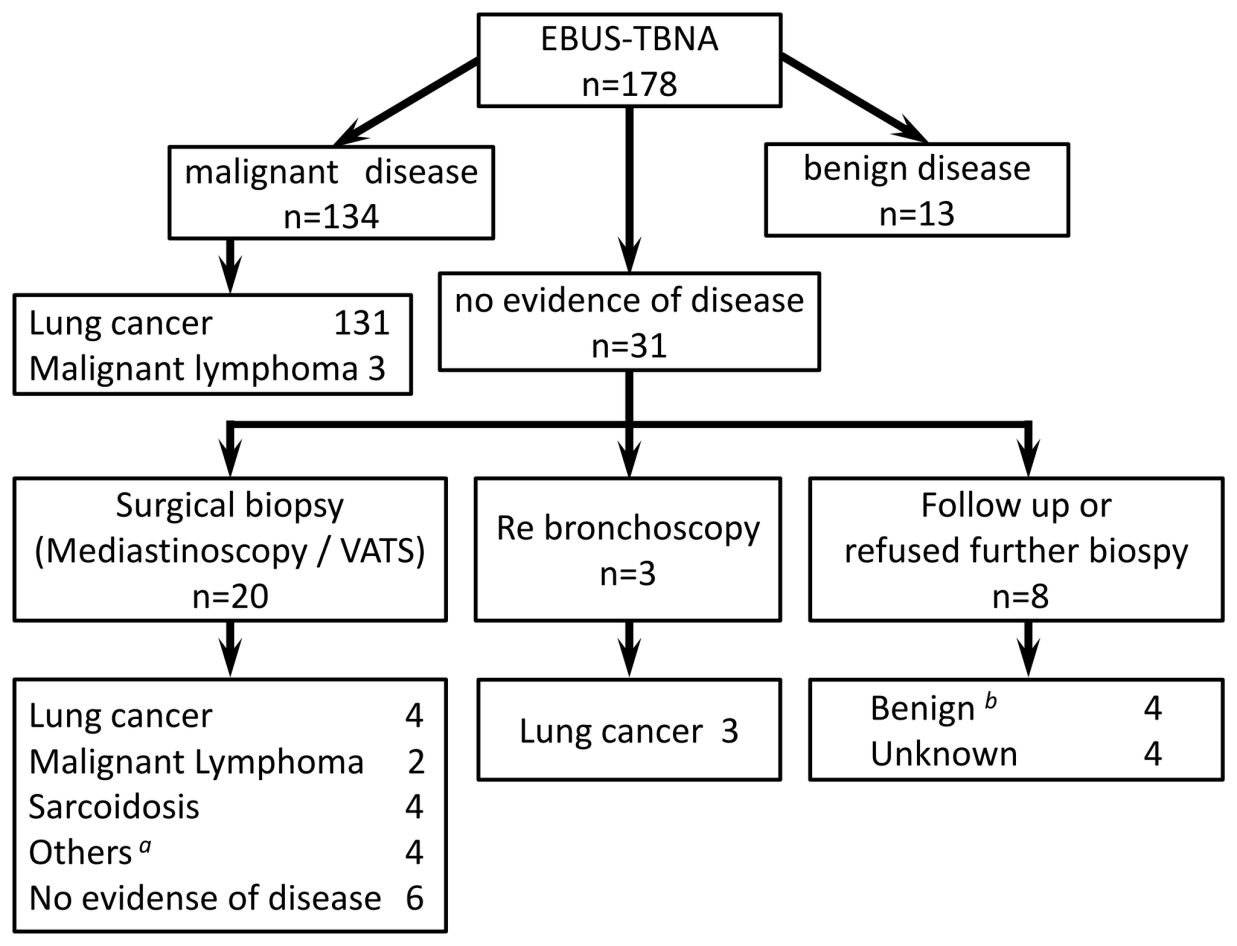
Flowchart of the 178 patients who underwent EBUS-TBNA. ^*a*^ Others included bronchogenic cyst, tuberculous lymphadenitis, inflammatory myofibroblastic tumor and leiomyoma. *^b^* The determination of benign diseases was based on results of clinical follow-up of at least 6 months demonstrating reduction in size or lack of apparent radiologic disease progression.

There were 178 patients who underwent EBUS-TBNA between April 2009 and March 2012. EBUS-TBNA detected malignancy in 134 patients and benign diseases in 13. The remaining 31 patients had no evidence of disease in specimens and therefore underwent surgical biopsy or transbronchial biopsy after EBUS-TBNA. Final diagnoses were malignant diseases in 143 cases and benign diseases in 31. Four patients were lost to follow up (Figure 2). Lung cancer was the most common malignancy (138/143, 96.5%) and sarcoidosis was the most common benign disease (6/31, 19.4%). 

Lung cancer was detected in 131 of 138 patients by EBUS-TBNA. Seven patients had non-diagnostic EBUS-TBNA specimens, and their lung cancer diagnoses were finally confirmed by surgery or transbronchial lung biopsy. Among the 131 patients with lung cancer diagnosed by EBUS-TBNA, 35 were in group C and 96 were in group H. The patient characteristics are summarized in Table 1. There were no significant differences in patient characteristics between the 2 groups. The sensitivity, specificity, and rates of diagnostic accuracy for differentiating malignant and benign disease by EBUS-TBNA were 92.3%, 100%, and 94.2% in group C and 94.2%, 100%, and 95.1% in group H, respectively (Table 2). There was no significant difference in diagnostic results between the groups (*p* = 1.0, Fisher’s exact test).

**Table 1:** Characteristics of patients who were initially diagnosed with lung cancer by EBUS-TBNA (n = 131).

	**Group C (n = 35)**	**Group H (n = 96)**	***p***
**Age**			0.07^*a*^
Median (range)	65(51-82)	69(42-87)	
**Gender**			0.46^*b*^
Female	5(14%)	20(21%)	
Male	30(86%)	76(79%)	
**Histology**			0.70^*b*^
Adenocarcinoma	11(31%)	40(42%)	
Squamous cell carcinoma	8(23%)	24^*c*^(25%)	
Small cell carcinoma	13(37%)	26(27%)	
Large cell carcinoma	1(3%)	2(2%)	
NSCLC- NOS	2(6%)	4(4%)	
**Location of LN station**			0.14^*b*^
2R	3(9%)	1(1%)	
4R	7(20%)	32(33%)	
4L	3(9%)	5(5%)	
7	12(34%)	35(37%)	
Others	10(28%)	23(24%)	
**LN stations per patient**			0.56^*b*^
1 station	32(91%)	82(85%)	
2 stations ≤	3(9%)	14(15%)	
**LN size^*d*^, mm, median (range)**			
Short axis	23.7(11.3-49.0)	20.9(10.1-39.4)	0.13^*a*^
Long axis	28.5(16.0-55.1)	27.2(13.2-49.2)	0.15^*a*^

*^a^* By Mann-Whitney *U* test. *^b^* By Fisher’s exact test. *^c^* Including 1 patient with combined squamous and small cell carcinoma. *^d^* Size of the largest LN station on computed tomography.

Abbreviations: NSCLC- NOS, Non-small cell lung carcinoma- not otherwise specified; LN, Lymph node; CT, Computed Tomography.

**Table 2:** Sensitivity, speciﬁcity, and rates of diagnostic accuracy of EBUS-TBNA.

	**Group C**	**Group H**
**Sensitivity**	92.3 %	94.2 %
**Specificity**	100 %	100 %
**Diagnostic accuracy rate**	94.2 %	95.1 %

There was no significant difference between group C and group H.

There were 7 patients in group C (7/35; 20%) and 6 in group H (6/96; 6%) who were diagnosed by cytology alone. There were 28 in group C (28/35; 80%) and 90 in group H (90/96; 94%) who were given diagnoses by both cytology and histology (Table 3). There was a significant difference between the groups in terms of the rate of sampling of sufficient histological specimens (*p* = 0.04, Fisher’s exact test). As for the amount of the tissue, there was no significant difference in the number of fragments between the two groups (median values, 13 in group C versus 10 in group H, *p* = 0.61), however, the total tissue area was significantly greater in group H (high pressure group) than in group C (median values, 2.1 × 10^5^ versus 4.8 × 10^5^ pixels, *p* = 0.003). There were no major complications, including infection or severe bleeding, related to the procedures in either group. 

**Table 3:** Diagnostic method according to group.

	**Group C**	**Group H**	***p***
**Diagnostic method**			0.04^a^
Cytology alone	7	6	
Cytology and Histology	28	90	

^a^ By Fisher’s exact test.

There was a statistically significant difference between group C and group H in terms of the rate of sampling of sufficient histological specimens.

## Discussion

Here we have compared different volumes of suction pressure during EBUS-TBNA biopsy in terms of sufficient tissue collection, particularly from mediastinal and hilar lymph nodes. EBUS-TBNA has been found to be an accurate and safe diagnostic technique for mediastinal and/or hilar lymphadenopathy and for staging of lung cancer. EBUS-TBNA is a real-time procedure that allows multiple biopsies with high-quality histologic cores [1–3]. It has been associated with only minimal complications. 

In past studies, the sensitivity and rates of diagnostic accuracy of EBUS-TBNA for differentiating malignant and benign disease has been reported from 85 to 93% and 88 to 91%, respectively [4–7]. In the present study, sensitivity and diagnostic accuracy were 93.7% and 94.8%, respectively, which were similar to the previous results. As for EBUS-TBNA approach, it is reported that 3 aspirations per lymph node station can be optimal [8], and there is no significant difference between results reported with 21- and 22-gauge aspiration needles [9,10]. However, little information about optimal suction pressure for extracting sufficient histological cores during EBUS-TBNA has been reported to date. Casal and colleagues [11] reported that there were no differences between samples collected with or without suction aspiration during EBUS-guided biopsy. However, they did not analyze the ability of each technique to provide histologic cores.

In this study, the main focus was the rate of sufficient core tissue sampling and not the diagnosis rate. We found that the rate of sufficient histological specimen sampling in group H (high pressure, 30-mL suction) was superior to that in group C (conventional method, 20-mL suction), and these results suggest that higher aspiration pressures during EBUS-TBNA may be useful for obtaining sufficient histological specimens and assisting with accurate diagnosis, including subclassification of lung cancers, as well as optimal treatment of patients with advanced and recurrent lung cancer. We emphasize that it is very important to obtain sufficient specimens when EBUS-TBNA is performed, not only for diagnosis but for additional studies including immunohistochemistry and genetic analysis, such as epidermal growth factor receptor (EGFR) and anaplastic lymphoma kinase (ALK). The proper EBUS-TBNA method permits sampling of histologic cores. The quantity of the acquired tumor cell is possibly one of the reasons for the higher rate of pathologic diagnoses in group H; however, further investigation is necessary to explore the most important factor contributing to higher rate of tissue core sampling.

Several recent studies comparing EGFR mutation status in primary tumor and local lymph node metastases have suggested a possibility of significant discrepancies between the sites because of tumor heterogeneity, which may be a cause of resistance to treatment with EGFR tyrosine kinase inhibitors [12–14]. Therefore, in cases of disease progression, repeat biopsy by minimally invasive techniques that allow molecular analyses should be considered. Our results indicate that EBUS-TBNA, especially with high suction pressure, may a procedure of choice.

There are several limitations of this study. First, it is a retrospective analysis in a single institute and it is not randomized. Second, the EBUS-TBNA procedures were not performed by the same bronchoscopist. In addition, patients in group C were biased around the introduction of EBUS-TBNA at our institute. Prior to the introduction of EBUS-TBNA, we trained bronchoscopists with a demonstration; however, the learning curve of the technique may affect the TBNA sampling rate. Third, the number of patients in this study was too small to definitively evaluate the optimal suction pressures for safe sampling during EBUS-TBNA. In order to do this, prospective trials will be required in the future.
